# Factors Associated with Aggravation of Esophageal Varices after B-RTO for Gastric Varices

**DOI:** 10.1007/s00270-013-0809-6

**Published:** 2013-12-10

**Authors:** Atsushi Jogo, Norifumi Nishida, Akira Yamamoto, Hiroto Matsui, Tohru Takeshita, Yukimasa Sakai, Toshiyuki Matsuoka, Kenji Nakamura, Yukio Miki

**Affiliations:** 1 Department of Radiology, Osaka City University Graduate School of Medicine, 1-4-3 Asahi-machi, Abeno-ku, Osaka, 545-8585 Japan; 2 IVR Center, Daito Central Hospital, Osaka, Japan

**Keywords:** Embolization, Portal vein hypertension, Transcatheter therapy, Varices, Venous intervention

## Abstract

**Purpose:**

To retrospectively evaluate risk factors for aggravation of esophageal varices (EV) within 1 year after balloon-occluded retrograde transvenous obliteration (B-RTO) of gastric varices (GV) and to clarify suitable timing for upper endoscopy to detect EV aggravation after B-RTO.

**Methods:**

Participants included 67 patients who underwent B-RTO for GV between January 2006 and December 2010. Whether EV aggravation occurred within 1 year was evaluated, and the time interval from B-RTO to aggravation was calculated. Factors potentially associated with EV aggravation were analyzed.

**Results:**

B-RTO was successfully performed in all patients. EV aggravation at 1 year after B-RTO was found in 38 patients (56.7 %). Multivariate logistic regression analysis showed that total bilirubin (T-bil) (*P* = 0.032) and hepatic venous pressure gradient (HVPG) (*P* = 0.011) were significant independent risk factors for EV aggravation after B-RTO. Cutoff values of T-bil and HVPG yielding maximal combined sensitivity and specificity for EV aggravation were 1.6 mg/dL and 13 mmHg, respectively. The patients with T-bil ≥ 1.6 mg/dL or HVPG ≥ 13 mmHg had a median aggravation time of 5.1 months. All five patients with ruptured EV belonged to this group. In contrast, patients with T-bil < 1.6 mg/dL and HVPG < 13 mmHg had a median aggravation time of 21 months.

**Conclusion:**

T-bil and HVPG were significant independent risk factors for EV aggravation after B-RTO. The patients with T-bil ≥ 1.6 mg/dL or HVPG ≥ 13 mmHg require careful follow-up evaluation, including endoscopy.

## Introduction

Gastric varices (GV) occur in 5–33 % of patients with portal hypertension [1–3]. Although the bleeding rate of GV is 5–25 %, lower than the rate of bleeding from esophageal varices (EV) [1, 4], the prognosis is worse than for EV once bleeding occurs, with a reported mortality rate of 45 % [1].

The concept of balloon-occluded retrograde transvenous obliteration (B-RTO) for GV was reported by Olson et al. [5], and the use of B-RTO was later expanded by Kanagawa et al. [6–8]. The treatment outcomes for GV are very good using this method, with recently reported bleeding rates after B-RTO of 2.7–3.2 % [9, 10]. However, worsening of EV and possible ascites due to portal hypertension after B-RTO are problems. Reportedly, the aggravation rate of EV was 27 % in the first year [9] and 66–67 % cumulatively [9, 11, 12]. Endoscopic examination for EV in cirrhotic patients has been recommended every 1–2 years [3]. Because early aggravation of EV is expected with worsening of portal hypertension after B-RTO, evaluation of risk factors for aggravation and the interval until the occurrence of aggravation are important. The purpose of this study was to retrospectively evaluate risk factors for aggravation of EV within 1 year after B-RTO and to determine when to perform upper endoscopy to detect aggravation of EV after B-RTO.

## Materials and Methods

### Patients

The ethics committee at our hospital deemed this retrospective study as appropriate for publication. The study initially included 164 consecutive patients who underwent B-RTO for GV between January 2006 and December 2010. Informed consent was obtained before the procedure. Treatment criteria for GV were as follows: (1) GV larger than F2 (moderately enlarged, beady varices, and/or red spot) as defined by the Japan Gastroenterological Endoscopy Society [13]; and/or (2) GV with diameter >5 mm on color Doppler endoscopic ultrasonography [14]; and/or (3) ruptured GV and primary hemostasis achieved. Treatment indications for B-RTO were a major portocaval shunt that, on the basis of preoperative computed tomography (CT), could anatomically be reached transvenously using a catheter, for example, by gastrorenal shunt, inferior phrenic vein, or pericardial vein. Sixty-seven patients were finally enrolled after excluding three patients who underwent selective B-RTO for preservation of a major portocaval shunt [15], nine patients who underwent scheduled treatment for EV during the same hospitalization, and 85 patients who were lost to follow-up (Table 1). Factors associated with aggravation of EV after B-RTO were statistically analyzed in these patients.

**Table 1:** Patient demographics

Characteristics	Value
Sex (M:F)	48:19
Age (years)	
Mean	67
Median (range)	66 (34–81)
Cause of cirrhosis	
Alcohol	16
Hepatitis B	5
Hepatitis C	35
Other	9
Biochemical data	67
T-bil (mg/dL)	1.4 ± 0.7
PT (%)	75 ± 13
Alb (mg/dL)	3.3 ± 0.5
Child–Pugh classification	
A	43
B	22
C	2
MELD score	
Median	62
Mean ± SD	9.7 ± 2.3
GV	
Lg-c	7
Lg-f	27
Lg-cf	33
F1	0
F2	36
F3	31
EV before B-RTO	
F1	30
F2	6
F3	0

Data provided as median (range); *n*; or mean ± SD

*T-bil* total bilirubin, *Alb* albumin, *PT* prothrombin time, *GV* gastric varices, *EV* esophageal varices, *B-RTO* balloon-occluded retrograde transvenous obliteration, *Lg-c* adjacent to the cardiac orifice, *Lg-cf* extending from the cardiac orifice to the fornix, *Lg-f* distant from the cardiac orifice, *F1* straight small-caliber varices, *F2* moderately enlarged beady varices, *F3* markedly enlarged nodular or tumor-shaped varices

### B-RTO Procedure

In patients with gastrorenal shunt as the main draining vein (*n* = 65), a 6F balloon catheter with an 11- or 20-mm diameter balloon (Moiyan; Miyano, Osaka, Japan) was inserted into this vessel to perform the procedure. In addition, on the basis of preoperative CT, for more selective insertion of the catheter near the varices, a 9F/5F double coaxial balloon catheter system (Candis; Medikit, Tokyo, Japan) was used [16]. In patients (*n* = 2) without gastrorenal shunt in whom the main draining vein was the pericardial vein or inferior phrenic vein, a microballoon catheter (Iiguman; Fuji Systems, Tokyo, Japan) was inserted in the draining vein.

In all cases, if the varices were adequately visualized with B-RTO, the sclerosing agent was slowly injected until the feeding veins were visualized under fluoroscopic guidance. The sclerosing agent consisted of 5 % ethanolamine oleate iopamidol (EOI) mixed with 10 % ethanolamine oleate (Oldamin; Takeda Pharmaceutical, Osaka, Japan) and the same volume of nonionic contrast medium (iopamidol 300 mg I/mL, Iopamiron 300; Bayer Schering Pharma, Osaka, Japan). If the varices were not visualized because of the presence of collateral draining veins, downgrading [17] was performed by embolization of the collaterals using a combination of 50 % glucose injection and coils or stepwise injection of 5 % EOI. The inflated balloon catheter was left in place overnight, and if thrombosis was confirmed the next day under fluoroscopy, the balloon catheter was deflated and removed. If thrombosis was insufficient, a sclerosing agent was added, thrombosis was reconfirmed after 6–7 h, and then the balloon catheter was deflated and removed. If a large amount of sclerosing agent had to be used, the procedure was completed the next day.

The definition of technical success was disappearance of blood flow from GV on color Doppler endoscopy and/or dynamic CT within 2 weeks after B-RTO.

### Definition of Aggravation for EV

Endoscopic findings of GV and EV were classified according to the criteria proposed by the Japanese Society for Portal Hypertension [13]. The form of varices was classified as follows: F1, straight small-caliber varices; F2, moderately enlarged, beady varices; and F3, markedly enlarged, nodular, or tumor-shaped varices. According to location, GV were classified as follows: adjacent to the cardiac orifice; distant from the cardiac orifice; or extending from the cardiac orifice to the fornix.

Aggravation of EV was defined on the basis of a comparison with endoscopy before B-RTO as worsening morphology, appearance of a red spot, development of new varices, or variceal rupture. Endoscopy was performed every 3–6 months after B-RTO. If anemia got worse or hematemesis occurred during observation, endoscopy was performed according to the circumstances. The number of days from time of B-RTO until aggravation of EV as confirmed by endoscopy was calculated, and whether aggravation of EV had occurred by the 1-year follow-up was evaluated.

### Evaluation of Ascites

The existence of transient ascites was judged by CT within 1 month after B-RTO. Refractory ascites was determined by outpatient clinic examinations in patients followed more than 6 months after B-RTO.

### Measurements of Drainage and Portal Vein Diameters

The diameter of the portal vein was estimated on contrast CT images at a point midway between the main bifurcation of the portal vein into the right and left main hepatic branches and the portal vein confluence. The diameter of the gastrorenal shunt was estimated from the short axis at the proximal side of the left renal vein [11].

### Pressure Measurement

A 5F balloon catheter (Cobra; Selecon MP catheter; Terumo Clinical Supply, Gifu, Japan) was inserted through the femoral vein, and pressures were measured using a manometer (Polygraph MSC-7000; Fukuda Denshi, Tokyo, Japan) [18]. The measured parameters were right atrial pressure, hepatic venous pressure, and wedged hepatic venous pressure. Hepatic venous pressure gradient (HVPG) was calculated as the difference between wedged hepatic venous pressure and free hepatic vein pressure. In addition, the changes in HVPG before and after balloon occlusion of the drainage vein was also measured.

### Statistical Analysis

All results are expressed as mean ± standard deviation (SD), median, or percentage. The rate of EV aggravation at 1 year after B-RTO was estimated in a univariate manner with Student’s *t* test and the *χ* ^2^ test using GraphPad Prism version 5.02 software (GraphPad Software, San Diego, CA) and in a multivariate manner using logistic regression with SAS for Windows version 9.3 (SAS Institute, Cary, NC). In all analyses, values of *P* < 0.05 were considered statistically significant. In univariate analysis, baseline status of age, sex, cause of cirrhosis, existence of EV or treatment history of EV before B-RTO, total bilirubin (T-bil) albumin, prothrombin time, sodium, platelets, Child–Pugh score, Model for End Stage Liver Disease (MELD) score, diameter of the drainage vein, diameter of the portal vein, HVPG, changes in HVPG, and volume of 5 % EOI were considered as covariates. In multivariate logistic regression, the baseline status of sex, cause of cirrhosis, T-bil, prothrombin time, diameter of drainage vein, HVPG, and volume of 5 % EOI were considered as covariates. The receiver operating characteristic (ROC) curve was drawn using JMP version 9.0.2 software (SAS Institute). The Youden index (sensitivity + specificity − 1) was used to select the optimal cutoff points on the ROC curves. The Kaplan–Meier method was used to estimate the median aggravation rate of EV after B-RTO, and the log-rank test was performed using GraphPad Prism software to compare Kaplan–Meier curves.

## Results

### Outcomes of B-RTO

B-RTO was successfully performed in all 67 patients (100 %). The mean volume of 5 % EOI used for B-RTO in 67 patients was 33.1 mL. Among 67 patients with GV treated with B-RTO, the median duration of endoscopy until aggravation of EV or last follow-up was 9.5 months (mean 11.7 ± 9.6 months, range 0.10–45.9 months). Aggravation of EV at 1 year after B-RTO was found in 38 patients (56.7 %), and the median aggravation time was 9.3 months. Five patients (7.5 %) experienced EV rupture after B-RTO, with times until rupture of 1.1, 1.6, 3.5, 4.6, and 9.3 months. All five patients underwent additional endoscopic treatment and were saved. All patients had EV or a treatment history of EV before B-RTO, and four of these patients had poor liver function, with a Child–Pugh class B or C. Ascites transiently developed or increased after B-RTO in eight of 60 patients (13.3 %). During a mean follow-up of 29.2 months (range 6–88 months) after B-RTO, refractory ascites was observed in one of 60 patients (1.7 %). This patient underwent endoscopic sclerotherapy for EV after 5 months and later underwent radiofrequency ablation for hepatocellular carcinoma. After 8 months, he underwent transjugular intrahepatic portosystemic shunt (TIPS) for refractory ascites.

### Evaluation of Risk Factors for Aggravation of EV after B-RTO

Sex, existence of EV, or history of treatment for EV before B-RTO, T-bil, prothrombin time, MELD score, diameter of drainage vein, HVPG, the changes in HVPG after balloon occlusion of the drainage vein, and volume of 5 % EOI were all identified by univariate analysis as significant risk factors for aggravation of EV after B-RTO. Other variables including age, cause of cirrhosis, albumin, sodium, platelets, Child–Pugh score, and diameter of the portal vein were not significant factors in univariate analysis (Table 2).

**Table 2:** Univariate analysis for factors associated with aggravation for EV after B-RTO

Variable	*n*	Mean (range)	EV aggravation	95 % CI	*P*
			(+)	(+)	
			(−)	(−)	
Age	67	66 (34–81)	63.4 ± 10.0	60.1–66.7	0.051^a^
			68.0 ± 8.7	64.7–71.3	
Sex (male vs. female)	67	47 vs. 20	31 vs. 7	NA	0.019^b^
			16 vs. 13		
Cause of cirrhosis (alcohol vs. others)	67	16 vs. 51	12 vs. 26	NA	0.091^b^
			4 vs. 25		
Existence of EV or treatment history of EV before B-RTO (presence vs. absence)	67	40 vs. 27	27 vs. 11	NA	0.044^b^
			13 vs. 16		
T-bil (mg/dL)	67	1.4 (0.4–3.7)	1.6 ± 0.7	1.4–1.9	0.0005^a^
			1.1 ± 0.5	0.9–1.2	
Alb (mg/dL)	66	3.3 (2.1–4.5)	3.2 ± 0.4	3.1–3.4	0.12^a^
			3.4 ± 0.5	3.2–3.6	
PT (%)	66	75 (38–102)	72.4 ± 14.0	67.8–77.0	0.032^a^
			79.5 ± 11.4	75.1–83.9	
Na (mEq/L)	60	141 (132–148)	138.9 ± 9.9	136–142	0.17^a^
			141.8 ± 2.3	141–143	
Plt (×10^4^/μL)	65	11.2 (2.3–67)	7.8 ± 3.6	6.6–9.0	0.15^a^
			11.0 ± 12.6	6.1–15.8	
Child–Pugh score	65	6.3 (5–10)	6.6 ± 1.5	6.0–6.9	0.18^a^
			6.0 ± 1.1	5.6–6.5	
MELD score	62	9.7 (6.4–16.9)	10.5 ± 0.4	9.7–11.3	0.0014^a^
			8.6 ± 0.6	8.0–9.3	
Diameter on CT					
Drainage vein (mm)	60	10 (5–22)	11.5 ± 4.4	9.9–13.0	0.021^a^
			9.0 ± 3.4	7.7–10.4	
Portal vein (mm)	60	12 (6–20)	12.3 ± 3.1	11.2–13.4	0.34^a^
			11.6 ± 2.5	10.6–12.6	
HVPG (mmHg)	46	13 (3–27)	14.5 ± 6.2	12.2–16.8	0.0007^a^
			8.2 ± 2.3	6.7–9.7	
Changes in HVPG	42	2.5 (0–9)	1.7 ± 1.7	1.0–2.3	0.022^a^
			4.2 ± 2.7	1.7–4.9	
Amount of 5 % EOI (mL)	63	33 (7–80)	37.1 ± 21.7	29.9–44.3	0.043^a^
			27.1 ± 13.2	21.6–32.5	

Data provided as median (range); *n;* or mean ± SD

*EV* esophageal varices, *B-RTO* balloon-occluded retrograde transvenous obliteration, *CI* confidence interval, *HVPG* hepatic venous pressure gradient, *EV* esophageal varices, *T-bil* total bilirubin, *Alb* albumin, *PT* prothrombin time, *Plt* platelets, *CT* computed tomography, *MELD* model for end stage liver disease, *EOI* ethanolamine oleate iopamidol

^a^Statistical comparisons performed by Student’s *t* test

^b^Statistical analysis was estimated by *χ* ^2^ test

**Fig. 1:**
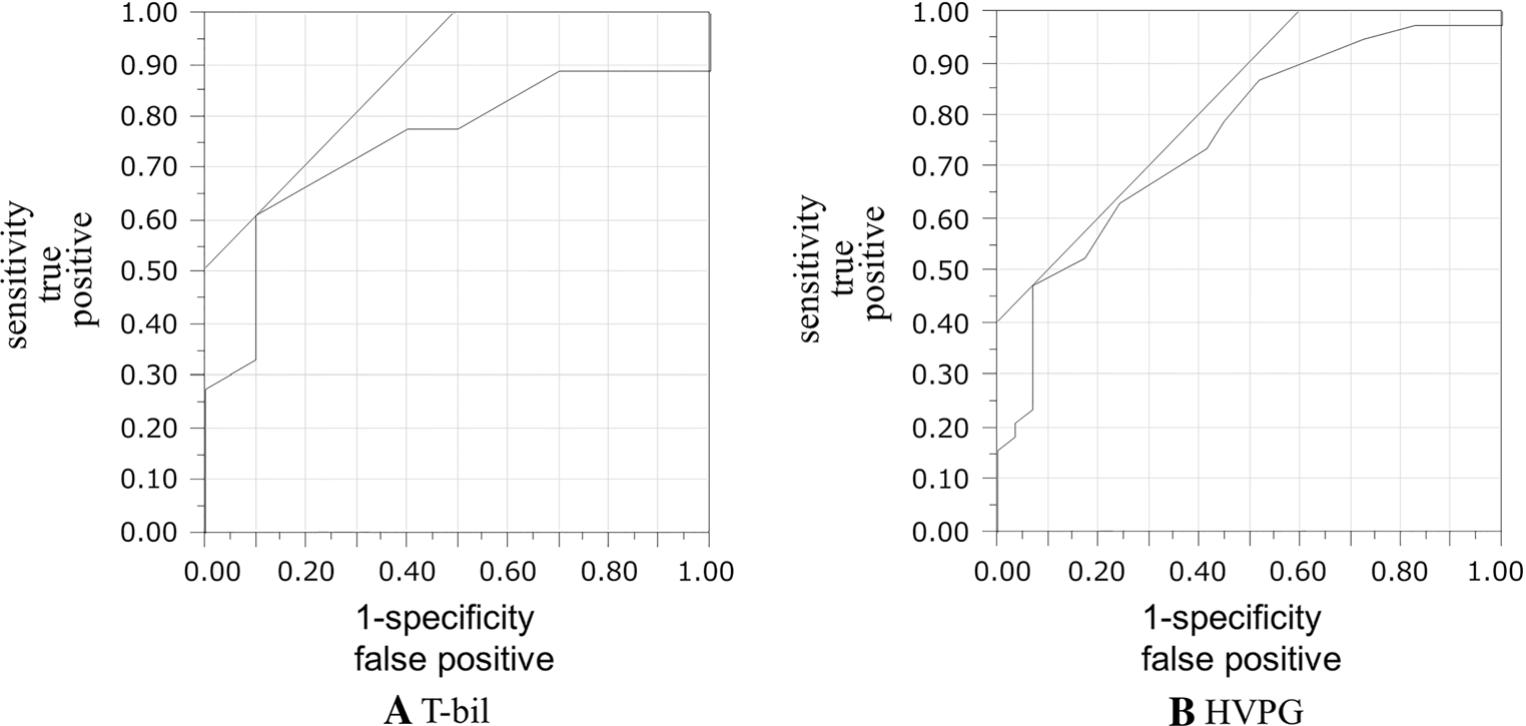
ROC curves for T-bil (**A**) and HVPG (**B**) were used to determine the cutoff values yielding the highest combined sensitivity and specificity with respect to aggravation of EV. Those points were 1.6 mg/dL for T-bil and 13 mmHg for HVPG, and areas under the ROC curve were 0.76 and 0.75, respectively. *ROC* receiver operating characteristic, *T-bil* total bilirubin, *EV* esophageal varices, *HVPG* hepatic venous pressure gradient

**Fig. 2:**
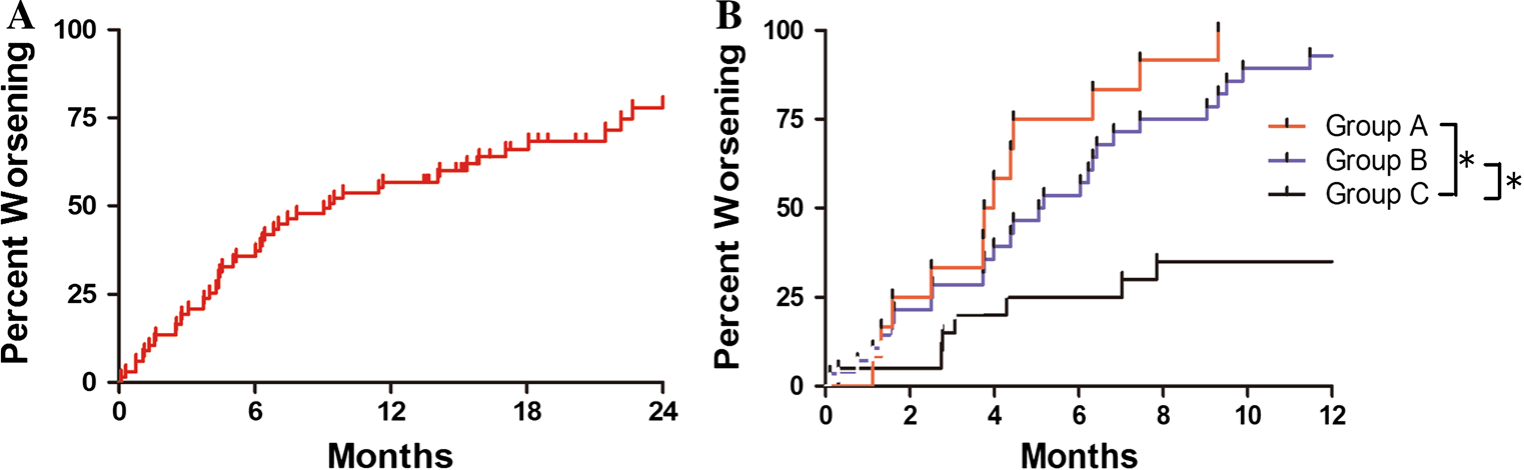
**A** Graph showing total aggravation rate of EV after B-RTO. Aggravation at 1 year was found in 38 of 67 patients (56.7 %), and median aggravation time was 9.3 months. **B** *Graph* showing aggravation rate of EV. We divided subjects into the following three groups according to cutoff values: group A, T-bil ≥ 1.6 mg/dL and HVPG ≥ 13 mmHg (*n* = 12); group B, T-bil ≥ 1.6 mg/dL or HVPG ≥ 13 mmHg (*n* = 25); and group C, T-bil < 1.6 mg/dL and HVPG < 13 mmHg (*n* = 7). Statistically, median aggravation time of EV after B-RTO was 3.8 months in group A, 5.1 months in group B, and 21 months in group C. Significant differences in aggravation time were observed between group A versus group C (*P* = 0.0001) and group B versus group C (*P* = 0.0002). In group A, all 12 patients experienced aggravation within 8 months. All five patients with ruptured EV belonged to group B. *EV* esophageal varices, *B-RTO* balloon retrograde transvenous obliteration, *T-bil* total bilirubin, HVPG hepatic venous pressure gradient, *MELD* model for end stage liver disease

All variables detected as significant by univariate analysis were then examined by multivariate analysis to identify independent significant factors. A logistic regression model using multivariate analysis showed T-bil (hazard ratio 83.3; 95 % CI 0.001–0.69; *P* for trend = 0.032) and HVPG (hazard ratio 0.011; 95 % CI 0.33–0.87; *P* for trend = 0.011) as independent significant risk factors for aggravation of EV after B-RTO (Table 3). In addition, ROC curves were used to determine the cutoff values of T-bil and HVPG yielding the highest combined sensitivity and specificity with respect to aggravation of EV. These values were 1.6 mg/dL and 13 mmHg, respectively. Areas under the ROC curve for T-bil and HVPG were 0.76 and 0.75, respectively (Fig. 1). Using these cutoff values, we divided these patients into following three groups: group A, T-bil ≥ 1.6 mg/dL and HVPG ≥ 13 mmHg (*n* = 12); group B, T-bil ≥ 1.6 mg/dL or HVPG ≥ 13 mmHg (*n* = 25); and group C, T-bil < 1.6 mg/dL and HVPG < 13 mmHg (*n* = 7). The median times to aggravation of EV after B-RTO were 3.8 months in group A, 5.1 in group B, and 21 in group C. A significant difference in aggravation time was found between groups A and C (*P* = 0.001) and groups B and C (*P* = 0.002) (Fig. 2B). In group A, all 12 patients experienced aggravation within 8 months. All 5 patients with ruptured EV belonged to group B.

**Table 3:** Multivariate analysis of aggravation factors for esophageal varices after balloon-occluded retrograde transvenous obliteration

Variable	HR	95 % CI	*P*
T-bil	82.4	1.46–>999.9	0.032
MELD score	0.75	0.28–0.76	0.58
HVPG	1.87	1.16–3.01	0.011

*HR* hazard ratio, *CI* confidence interval, *T-bil* total bilirubin, *MELD* model for end stage liver disease, *HVPG* hepatic venous pressure gradient

## Discussion

The mechanism of B-RTO-related aggravation of EV involves changes in hemodynamics, including changes in pressure and blood flow associated with shunt occlusion [11, 19]. Our results of univariate analysis showed that sex, history of EV or treatment for EV before B-RTO, T-bil, prothrombin time, MELD score, draining vein diameter, HVPG, changes in HVPG, and volume of 5 % EOI were significant risk factors. Multivariate analysis identified T-bil and HVPG as independent factors significantly associated with aggravation.

Endoscopic examination for EV in cirrhotic patients has been recommended every 1–2 years [3]. However, the optimal follow-up period with endoscopy after B-RTO has not yet been reported. In our study, the median time to aggravation of EV in patients with T-bil ≥ 1.6 mg/dL and HVPG ≥ 13 mmHg was 3.8 months, and all those patients showed aggravation of EV within 8 months. The patients with T-bil ≥ 1.6 mg/dL or HVPG ≥ 13 mmHg showed a median aggravation time of 5.1 months. Moreover, all patients with ruptured EV after B-RTO satisfied these conditions. These types of patients require careful follow-up evaluation, including endoscopy at shorter follow-up intervals. An optional therapy such as selective B-RTO or addition of TIPS after B-RTO might prevent excessive worsening of portal hypertension [20]. In some high-risk patients, TIPS alone or no treatment might be a therapeutic option. On the other hand, patients with T-bil < 1.6 mg/dL and HVPG < 13 mmHg had a median aggravation time of 21 months. The aggravation risk of EV was considered to be relatively low in these patients.

Higher T-bil values before B-RTO were associated with significant aggravation of EV after B-RTO, with a cutoff value of 1.6 mg/dL. Scheig [21] and Malinchoc et al. [22] reported bilirubin as one of the better liver function tests because the liver must take bilirubin away from the albumin to which it is bound in the circulation, conjugate it, and excrete it into the bile, thus representing a truly complete series of reactions. In a study of 39 patients, Elsamman et al. [11] found that a higher Child–Pugh class was associated with aggravation of EV after B-RTO. Currently, albumin level, encephalopathy, and ascites can be altered by medical intervention, including administration of branched-chain amino acids, Zn preparations, and diuretics [23]. These may be one of the reasons why the Child–Pugh score was not significantly associated with aggravation in this study, though the patients with ruptured EV after B-RTO had mostly poor liver function before B-RTO [24, 25]. In univariate analysis, the MELD score was a risk factor while the Child–Pugh score was not. The MELD score reflects survival after TIPS in end-stage liver disease [22, 26, 27]. This model is superior to the Child–Pugh score in predicting survival [22]. It uses renal function because renal dysfunction carries a poor prognosis. In general, renal function is not directly associated with aggravation of EV. This may be one of the reasons why T-bil outperformed MELD in our multivariate analysis.

Higher HVPG values before B-RTO were also associated with significant aggravation of EV after B-RTO, with a cutoff value of 13 mmHg. Portal pressure has been shown to correlate closely with severity of liver cirrhosis, as assessed by liver biopsy [28, 29]. Silkauskaite et al. [30] reported that HVPG also correlates with severity of liver disease, size of varices, and bleeding status. Garcia-Tsao et al. [31] reported that HVPG >12 mmHg is necessary for the occurrence of variceal hemorrhage and for the appearance of gastroesophageal varices. The changes in pressure from before to after B-RTO have also occasionally been reported. In a study of 20 cirrhotic patients, Tanihata et al. [18] reported that a ≥5 mmHg increase in the portal systemic pressure gradient (PSPG) after B-RTO was a factor associated with aggravation of EV. On the other hand, in a study of 24 cirrhotic patients, Hayashi et al. [32] found no significant changes in wedged hepatic venous pressure or HVPG after B-RTO. In our study, changes in HVPG were not significant in multivariate analysis. Although there is still room for discussion, our findings showed that HVPG before B-RTO. In other words, baseline portal pressure had an impact on aggravation of EV after B-RTO.

Univariate analysis showed that a history of EV or treatment for EV before B-RTO was significantly associated with aggravation. The presence of EV on endoscopy before B-RTO as a significant aggravation factor for EV after B-RTO has occasionally been reported [9, 11]. Higher F stage of GV before B-RTO also tends to be an aggravating factor for EV after B-RTO [33]. Moreover, in a study on the hemodynamics of extrahepatic collaterals using portography from the superior mesenteric artery before B-RTO, patients with a higher number of collateral routes such as a paraesophageal vein, compared to a gastrorenal shunt or gastric-inferior phrenic vein shunt alone, displayed a significantly higher rate of EV aggravation after B-RTO [32]. Our findings in this study are in general agreement with those reports.

In addition, as the draining vein diameter became larger or the volume of EOI used increased, EV showed significantly higher risk of aggravation. Draining vein diameter or volume of EOI used may reflect the volume of the embolized area. If the volume of the embolized area is large, the volume of interrupted blood flow is also larger, and EV may thus be aggravated.

The rate of EV aggravation in the first year after B-RTO in our study was high, at 56.7 %. Differences in the rate of EV aggravation after B-RTO have been reported in recent studies, ranging from 17 to 63 % [9–11, 18, 33, 34]. This is due to differences in how aggravation of EV is defined and in the duration of follow-up. Some reports have defined EV aggravation as when “varices become enlarged, tortuous, or large and coil shaped, or when a red spot is observed” [18, 33, 34]. On the other hand, other reports have defined EV aggravation as when “red spots on EV and/or bleeding of EV is detected” [9–11].

Development of refractory ascites after B-RTO may become an issue [20]. We experienced only one patient who developed refractory ascites after B-RTO in 67 patients. Furthermore, the patient underwent treatments for EV and hepatocellular carcinoma, which might be also related to development of refractory ascites. In this study, the follow-up time was relatively short (mean, 26 months). Further study of refractory ascites is needed.

Various limitations must be considered when interpreting the results of the present study. These include the retrospective nature of the study and a follow-up interval for endoscopy ranging from 3 to 6 months. Eighty-five patients were lost to follow-up, which may affect the results. In addition, patients treated for EV during the same hospitalization were excluded; such cases may have involved early aggravation of EV after B-RTO.

In conclusion, T-bil levels and HVPG were identified as independent risk factors for aggravation of EV at 1 year after B-RTO. The patients with T-bil ≥1.6 mg/dL or HVPG ≥13 mmHg had median aggravation time of 5.1 months after B-RTO. These types of patients require careful follow-up evaluation, including endoscopy.
